# Older Adults’ Experiences With Using Technology for Socialization During the COVID-19 Pandemic: Cross-sectional Survey Study

**DOI:** 10.2196/28010

**Published:** 2021-04-23

**Authors:** Kristen R Haase, Theodore Cosco, Lucy Kervin, Indira Riadi, Megan E O'Connell

**Affiliations:** 1 School of Nursing Faculty of Applied Science University of British Columbia Vancouver, BC Canada; 2 Gerontology Research Center Department of Gerontology Simon Fraser University Vancouver, BC Canada; 3 Oxford Institute of Population Ageing University of Oxford Oxford United Kingdom; 4 Department of Gerontology Simon Fraser University Vancouver, BC Canada; 5 Department of Psychology University of Saskatchewan Saskatoon, SK Canada

**Keywords:** older adults, social isolation, COVID-19, technology use, eHealth

## Abstract

**Background:**

Technology use has become the most critical approach to maintaining social connectedness during the COVID-19 pandemic. Older adults (aged >65 years) are perceived as the most physiologically susceptible population to developing COVID-19 and are at risk of secondary mental health challenges related to the social isolation that has been imposed by virus containment strategies. To mitigate concerns regarding sampling bias, we analyzed a random sample of older adults to understand the uptake and acceptance of technologies that support socialization during the pandemic.

**Objective:**

We aimed to conduct a population-based assessment of the barriers and facilitators to engaging in the use of technology for web-based socialization among older adults in the Canadian province of British Columbia during the COVID-19 pandemic.

**Methods:**

We conducted a cross-sectional, population-based, regionally representative survey by using the random-digit dialing method to reach participants aged >65 years who live in British Columbia. Data were analyzed using SPSS (IBM Corporation), and open-text responses were analyzed via thematic analysis.

**Results:**

Respondents included 400 older adults aged an average of 72 years, and 63.7% (n=255) of respondents were female. Most respondents (n=358, 89.5%) were aware of how to use technology to connect with others, and slightly more than half of the respondents (n=224, 56%) reported that, since the beginning of the pandemic, they used technology differently to connect with others during the pandemic. Additionally, 55.9% (n=223) of respondents reported that they adopted new technology since the beginning of the pandemic. Older adults reported the following key barriers to using technology: (1) a lack of access (including finance-, knowledge-, and age-related issues); (2) a lack of interest (including a preference for telephones and a general lack of interest in computers); and (3) physical barriers (resultant of cognitive impairments, stroke, and arthritis). Older adults also reported the following facilitators: (1) a knowledge of technologies (from self-teaching or external courses); (2) reliance on others (family, friends, and general internet searches); (3) technology accessibility (including appropriate environments, user-friendly technology, and clear instructions); and (4) social motivation (everyone else is doing it).

**Conclusions:**

Much data on older adults’ use of technology are limited by sampling biases, but this study, which used a random sampling method, demonstrated that older adults used technology to mitigate social isolation during the pandemic. Web-based socialization is the most promising method for mitigating potential mental health effects that are related to virus containment strategies. Providing telephone training; creating task lists; and implementing the facilitators described by participants, such as facilitated socialization activities, are important strategies for addressing barriers, and these strategies can be implemented during and beyond the pandemic to bolster the mental health needs of older adults.

## Introduction

The potential impact of the global COVID-19 pandemic has been thought to have devastating implications for older adults, who have a high risk of developing COVID-19. Older adults have been experiencing social isolation as a result of physical distancing, which may lead to long-term mental health problems [1,2]. Early reports from China [3] have suggested that mental health support should be integrated into pandemic planning. However, nearly 1 year into the pandemic, such support has yet to be realized. Early in the pandemic, global calls were issued to address the risks of social isolation for older adults, which can be mitigated by using technologies that help reduce social isolation in times of physical distancing and address vital socialization functions [4,5]. Rapid funding calls were issued by health research agencies to determine how to best address and support the mental health needs of older adults [6].

Many technologies are available for mitigating the mental health consequences of social disconnection that has resulted from the COVID-19 pandemic [5]. A growing number of older adults have been adopting technology [7], and technology-facilitated social connection interventions have a strong evidence base for improving the mental and physical health of older adults [8-12]. However, to date, much of this evidence has come from self-selecting samples of older adults who are recruited via convenience sampling and participant members of community groups [13]. These groups may not provide accurate cross-sectional data on older adults’ use and uptake of technology at the population level. This is particularly true for nonrandomized studies of technology and older adults. It is likely that self-selecting participants disproportionately represent people from higher socioeconomic strata and younger age groups, those with higher educational levels and greater digital literacy, and those who prefer in-person communication over web-based platforms [14].

In our previous study [O’Connell ME, unpublished data, 2021], which was related to the pandemic, we hypothesized that pandemic-created conditions have resulted in technology becoming essential tools for meeting the socialization needs of older adults. We found that older adults value one-on-one remote telephone mentoring and the use of structured task-lists for guiding their engagement with web-based socialization activities. However, to understand whether web-based socialization is acceptable to a population-based sample of older adults, more research is needed.

The purpose of this study is to conduct a province-wide survey in the Canadian province of British Columbia and to understand the barriers and facilitators to engaging in web-based socialization activities among older adults. This will complement our existing British Columbia–based study, which involves an environmental scan of web-based socialization programs and in-depth interviews with older adults who engage with and disengage from web-based socialization activities during the pandemic.

## Methods

### Recruitment

In January 2021, we conducted a population-based, cross-sectional survey with adults aged >65 years. The survey was conducted by the Canadian Hub for Applied and Social Research (CHASR). We used a random-digit dialing approach to call landlines and mobile phones within the province of British Columbia. Upon providing consent to participate, individuals were asked to confirm that they were aged >65 years and resided within the province before they proceeded to answer the substantive and sociodemographic questions. Sociodemographic questions involved the following topics: gender, the highest level of education, and household income. The survey questions were intended to obtain a snapshot of technology use during the pandemic, which included aspects such as any changes to technology use, barriers and facilitators, and how technology was used to mitigate social isolation during the COVID-19 pandemic. The survey was piloted by CHASR staff among a random sample of five older adults to ensure that questions were acceptable and understandable to participants. The specific survey questions are shown in Textbox 1.

**Survey questions.**

1. Are you aware of how you can use technology to connect with others?
2. Since the beginning of the pandemic, are you using technology differently to connect with others?
3. Was this new technology or had you used it before?
4. Are you still using technology or did you stop?
5. What stops you from using technology to connect with others?
6. What would help or has helped you use technology to connect with others?
7. Are finances a barrier to your use of technology?

### Data Analysis

Data analyses were performed using SPSS, version 27.0 (IBM Corporation). Descriptive statistics, including means and frequencies, were used to describe the study sample.

### Thematic Analysis

Of the 7 questions, 2 were used to obtain open-text data, and we sought to thematically analyze these data to understand the main barriers and facilitators to using technology. We used a qualitative approach to open-text survey analysis, as per Braun et al [15]. For both questions 5 and 6 (Textbox 1), three authors (KH, LK, IR) conducted a thematic analysis. Responses were read, reread, coded with a descriptive label, and organized into broad themes. Themes and exemplar quotes were reviewed by the team to ensure consensus on the content and nature of the themes.

## Results

### Characterization of the Sample

Participants were aged an average of 72.2 years (range 65-107 years; SD 7.38 years), and 63.7% (255/400) of the sample were female. The majority of participants (264/400, 66%) completed some level of postsecondary education; 21.5% (85/400) of participants completed or took some technical or college courses, 28.8% (115/400) took some or completed university courses, and 15.8% (63/400) completed postgraduate training (eg, Master’s, professional, or doctoral degree). The remaining participants completed some (44/400, 11%) or all of high school (91/400, 22.8%). Of the 248 participants who reported their total household income from all sources, including pensions, 25 (10%) reported an income of less than Can $25,000/year (US $19,858/year), 74 (29.8%) reported an income that ranged from Can $25,000/year to Can $75,000/year (US $19,858/year to US $59,574/year), and 55 (22.1%) reported an income of over Can $75,000/year (US $39,574/year).

### Using Technology to Connect With Others

The majority of respondents (358/400, 89.5%) stated that they were aware of how to use technology to connect with others. Since the beginning of the pandemic, slightly more than half of the participants (224/400, 56%) reported that they used technology differently to connect with others. Furthermore, 55.9% (223/400) of respondents reported that they adopted new technology since the beginning of the pandemic. Age was not associated with using technology differently during the pandemic (point-biserial correlation coefficient [*r_pb_*]=−0.09; *P*=.089), but increasing age was associated with fewer people reporting that they knew how to use technology to connect with others (*r_pb_*=−0.21; *P*<.001). Compared to those with lower education, participants with higher education were more aware of how to use technology to engage with others (Spearman rank correlation coefficient [*r_s_*]=−0.16; *P*=.002) and were more likely to report that the pandemic changed the way that they used technology (*r_s_*=−0.17; *P*=.001).

### Barriers to Web-Based Socialization

In total, 91% (364/400) of participants reported that they continued to use the technologies that they started using during the pandemic. The remaining 9% (36/400) reported that they stopped using technology. The reasons for stopping the use of technology or not using technology varied. In total, 53 respondents stated that they did not use or stopped using technology during the pandemic. The three main reasons for not using technology included the following: (1) a lack of interest; (2) a lack of access; and (3) physical limitations. These barriers are described below.

Participants’ lack of interest was reported to by a result of their disinterest in computers in general, preferences for and comfort with the use of telephones, and feelings of simply being “too old.” One respondent’s lack of interest in computers was described as follows:

I like to do other things and I've never been that way inclined. It came out when I was too old to be bothered with it.

Another respondent said, “I’m old school and I don’t need it.” Others explained that when they retired, they did not want to deal with computers anymore. For instance, one participant stated:

When I retired, I said, “I don't want to look at another one for years. I have other things to do.”

Participants also stated that their affinity for using the telephone arose because telephones were their main means of connection. One participant simply stated, “I would rather talk to people on the phone*.*” Others described their desire to hear a human voice and felt that this was impossible to achieve with other forms of technology. A participant said:

I watch a lot of young people who do not socialize the way I like to socialize. I would rather communicate with people over the phone over text messages. I do not choose to do anything online; I am computer illiterate.

A final quote from one woman indicated how personal disinterest can drive one’s aversion to technology. She stated:

I do not like technology at all, and people should use their minds and not technology. Old ladies do not like it. I don't do gadgets.

A lack of access to technology for socialization included barriers such as financial costs, a lack of trust, and a lack of knowledge. Several older adults stated that they simply “don’t have a computer” and that this prevented them from using computers for socialization. Others felt the costs of either technology or the internet was “too expensive.” For example, one respondent simply said their main barrier was “not having the internet because it’s too expensive; [I] can't afford it.” Others described a lack of knowledge as an insurmountable challenge. For instance, a respondent said, “I have no idea about anything.” Another respondent expressed a newfound desire to learn about using technology since the start of the pandemic but felt that they did not have the skills to do so. This respondent stated:

I don't feel good at it and never had the need until last year and I wish I had done it.

Several participants felt that although their lack of knowledge was a problem, they would use technology if training was more accessible. A respondent stated that they would use technology “if there had been an affordable course for education.” They went on to say:

I started using computers in 1969, but I didn't keep up. If there was a course for other idiots like me, I would be more aware. But I have looked, and it is too expensive to pay for a weekend course.

The final barrier was related to those who were physically unable to use technology for web-based socialization, which included five individuals. One of the physical challenges described included eyesight (“I have bad eyesight and it is worse to see things; it is difficult”). Furthermore, two individuals believed that their experiences of having a stroke impacted their ability to engage with technology. One stated:

I have had a stroke and only have my left hand with which to type, so I haven't done any typing since that stroke. It is intimidating.

Two respondents experienced cognitive challenges resulting from a head injury, and one person reported “cognitive impairment” as a primary barrier that impeded their use of technology.

### Facilitators to Web-Based Socialization

We also asked participants to describe what helped them or would help them use technology for socialization during the pandemic. In total, 91.5% (366/400) of respondents provided at least 1 facilitator to technology use. The themes that described key facilitators were related to the following: (1) prior knowledge of technology; (2) the act of asking others for help; (3) technological accessibility; and (4) social motivation.

Respondents believed that prior knowledge of and familiarity with technology was fundamental to their ability to use technology to socially connect with others during the pandemic. Many participants indicated that learning how to use specific technologies helped them to stay connected with others during the pandemic. One respondent stated:

I guess learning how to use it and people guiding us along, do this do that here's how to mute, hints that people have given through the months.

Many older adults had prior knowledge of technology use. One respondent said:

I go to work and use a lot of technology in my work.

Other respondents obtained technology knowledge by seeking answers and instructions from web-based platforms. One person stated that they were familiar with technology and adopted new tools to connect with others since the pandemic began. This person said:

I've been using the internet for years. Zoom was new to me. My husband has used it before. It wasn't new, but I hadn't used it personally. We use it now to connect without any problems.

Several participants also described how they used their limited technology skills to learn more by simply searching the internet. One participant said that they “just [found] information on the internet on how to do it.”

Many older adults felt they were not capable of independently using technology and heavily relied on access to help, which typically came from family members or friends when the need to use technology arose. A respondent said:

If I have a question, I can ask [my kids]. They're my tech support.

Another respondent stated:

My son, who was a computer expert, helps me Skype so I can see my friends in a different province.

Participants also depended on their friends—fellow older adults—to adopt new technologies during the pandemic. One participant said:

A friend of mine introduced me to using my iPad, and if I have a problem, I phone her.

Participants emphasized the importance of having access to the support they needed to use technology, especially during a pandemic, as technology was their only connection to people. One participant stated:

When I have problems with my computer or iPad, my son is the one who would come and troubleshoot it for me. Email has helped me and Zoom as well. I am retired, so I just email or telephone them to have a one-on-one conversation. I find it really important right now to have a one on one in this time of virus with our technology, such as phone or email.

Several older adults also indicated that they put little time and effort into maintaining or using technology and preferred having someone facilitate their technology experiences. For instance, a participant stated:

When I do go online with Zoom, I rest in the comfort of somebody else facilitating it.

Older adults’ perceptions of the accessibility and user-friendliness of technological devices, services, and platforms considerably determined their willingness to use technology to socially connect with others. Older adults repeatedly voiced a desire for clearer instructions (than those that are typically available) on how to use technologies and technology applications. They also described the need for or use of supportive technology services that facilitate their technology use. One respondent stated:

I'm not very comfortable using Zoom. It is difficult to connect. I can use other things, but experiences with Zoom or group meetings is very confusing and has been cutting out. it could be clearer and easier to connect. I am older but not isolated from tech.

Access to up-to-date technologies and devices, such as smartphones, and the availability of reliable internet connectivity were additional facilitators of older adults’ engagement with technology. One participant said:

Well, more education on different modern stuff. Things like newer cellphone, cameras, and etc. We don’t use latest technology. When you look at my date of birth, you’ll see that I’m no youngster.

Several respondents believed that their use of technology was enabled or obstructed by their environment as a result of both social and material factors. Older adults who lived in rural or isolated communities experienced heightened barriers to technology use. Additionally, older adults who remained in the workforce reported a heightened use of and increased access to technology for work-related activities. A respondent stated that a main facilitator was “the fact that I am still working.”

Older adults also believed that their engagement with technology for connecting with others was largely driven by social motivation. A participant stated:

I am doing it too because everyone is doing it: social interest.

This motivation has arisen not only due to a desire to maintain social connections that were established prior to the pandemic, but also due to social pressures to use technology, as older adults’ friends, family members, and wider social circles and communities have increasingly used web-based platforms as a result of pandemic-related restrictions. One participant described this social pressure and its related implications for learning how to use technology for web-based socialization. This participant stated that “this technology was used by my peers and I had to learn it.”

## Discussion

### Principal Results

The 400 older adults we surveyed were aware of how to use technology to connect with others and reported that, since the beginning of the pandemic, they used technology differently to connect with others during the pandemic. More than half of the respondents (223/400, 55.9%) reported that they adopted new technology since the beginning of the pandemic. Nevertheless, older adults reported several key barriers to using technology, such as a lack of access, a lack of interest, and physical barriers. However, participants described more facilitators than barriers. Such facilitators included personal knowledge, support from family and friends, and the social pressures imposed by the pandemic. These findings are promising as we enter the second year of the pandemic. Although there have been optimistic plans for vaccinating the majority of Canadians within the year, it is unclear whether such plans will be realized. A clearer understanding of the use of web-based socialization technology among older adults will provide important opportunities for identifying future interventions and support services during and beyond the pandemic.

### Limitations

The sample in this cross-sectional study was predominantly female and more well educated than the general population. Therefore, our sample may not be representative of the general population. Furthermore, in an effort to keep the survey brief (as it was conducted via telephone), we only included a minimal number of demographic-related questions. In ongoing research, we intend to closely examine the individual situations of older adults in greater depth. Although we used a random sampling approach and were able to gather the perspectives of individuals who did and did not use technology (which is a challenge in the majority of technology-related research), our results are influenced by an element of response bias. We were unable to report a true response rate due to the limits of random-digit dialing, as the overall number of refusals could not be tracked with certainty. For example, it was unclear whether phone hang-ups were actually from households with eligible participants (adults aged >65 years).

### Comparison With Prior Work

The implications of social isolation and the reduced number of social networks were well documented prior to the pandemic [16]. However, our findings show that many older adults are doing their best to obtain family and community support to access technologies and demonstrating many strengths during the pandemic. These findings are in line with prior qualitative work by the first author, wherein older adult cancer survivors described their previous life and illness experiences of coping with the pandemic [17,18]. Drawing further information from older adults’ interests, motivations, and willingness with regard to their engagement with technology has promising implications for mitigating the devastating effects of the isolation that has been imposed by the pandemic.

In prior and ongoing work that was conducted during the pandemic, we studied the landscape of web-based socialization and the potential of remote technology training and mental health support. This survey study mirrors our prior study of a small sample of older adults, in which structured task lists were seen as integral in supporting older adults’ use of technology to meet their web-based socialization needs [O’Connell ME, unpublished data, 2021]. Furthermore, our population-based survey study demonstrated that clear instructions for using technologies, such as Zoom, for socialization purposes are needed for older adults to feel confident in their use of such technology. Our findings also support the potential of partnering with community groups that provide technological support (eg, task lists) and implementing remote training for using technology to encourage technology use among older adults.

In this study, the fact that several respondents did not feel confident in their technology use is an important implication. A lack of exposure to technology (ie, the digital divide) can result in additional psychological barriers to technology use (ie, the double digital divide). For example, participants from rural areas are unlikely to have access to the physical infrastructures that are needed for facilitating exposures to technology. This adds a psychological, technology adoption–related barrier to the existing technology adoption barriers that are described in the rural technology acceptance model [19]. The psychological barriers to technology adoption include those that were revealed in our study, such as the low perceived ease of use of new technologies, which was noted in the technology accessibility theme. A more fundamentally important psychological barrier to technology adoption may be the perception that technology is not going to be useful [19]. Data from O’Connell et al [19] are in line with those of other studies; the COVID-19 pandemic has changed how older adults perceive technology’s usefulness. They now see technology use as a method for maintaining social and community ties while complying with physical distancing measures and have therefore increased their adoption of technology. In a previous study, we described how remote training and support can help facilitate technology adoption, which would help people maintain social and community connections during the pandemic [O’Connell ME, unpublished data, 2021]. We also reported how the process of the pandemic’s impact on older adults’ perceptions of technology is described in the COVID-19–technology acceptance model [O’Connell ME, unpublished data, 2021].

In terms of addressing the challenges of the digital divide, incorporating the perspectives of older adults is an integral aspect of the increased use and uptake of digital technologies [20]. The first step in achieving positive, technology-driven outcomes in mental health (and other fields) is the articulation of barriers and facilitators. By conducting studies that examine older adults’ perspectives via exploratory qualitative analyses, we were able to better understand older adults’ perceived needs, challenges, and benefits in the context of evolving technologies. The cocreation of technologies with older adults has been suggested as an effective means of increasing technology engagement among older adults with mental health conditions [21].

We found that older adults’ engagement with technology may be impeded or facilitated by a range of factors, and this finding is in line with those of related, prepandemic research. Our results were similar to the findings of previous studies that examined older adults’ views on using technology to maintain social connectedness before the pandemic. Széman [22] has noted that older adults prefer to use simple software and websites. This implies that the difficulty and complexity of new technologies have always prevented older adults from using such technology to connect with others. On the other hand, our findings indicate that older adults can learn how to effectively use technologies to build or maintain social connections, especially when they experience the social pressure to do so [23]. Prior research has found that feelings of fear and resentment toward using technology can be overcome by training and increasing the frequency of use, as people gain confidence as they continue to use technological devices and navigate through websites and social media platforms [24].

Familial support and the drive to maintain family relationships have previously been identified as key facilitators of technological engagement among older adults. In our study, older adult participants used technology as a means of maintaining contact with close family members, and this finding is similar to those of previous studies [22,25]. These studies have indicated that engagement and communication with family members, especially with grandchildren, are driving factors for participants’ willingness to learn about new technologies. Notably, many previous studies that implemented technological interventions also provided either in-person or remote support to older adult participants to provide assistance or ensure that applications were operating in the correct manner [26,27]. In such studies, interventions that were not supported by research staff relied on support from family members [28]. This calls attention to the importance of older adults’ access to support while they learn how to use new technologies, regardless of whether this support comes from family or external sources. This was made evident by the frequency with which our respondents described how they relied on friends, family members, or supportive services to facilitate or provide instructions on the use of technology for social engagement.

Studies similar to ours have found that physiological barriers, such as hearing and vision loss [29] or issues with dexterity and poor coordination [25,30-32], may impede older adults’ willingness to engage with technology. This sentiment was also reported by our respondents, who stated that physical or cognitive limitations related to injuries or age-related decline inhibited their use of technological applications.

### Conclusions

Older adults are facing an unprecedented challenge during the COVID-19 pandemic. This study provides information on the facilitators of accessing technology that supports socialization and the barriers that are the most important to older adults. Our findings regarding these barriers and the mounting evidence in existing literature demonstrate that partnerships with community groups can potentially bolster socialization support for older adults. These study findings have immediate implications for supporting older adults. However, our findings’ sustained importance will be determined by how we can facilitate the use of technologies for socialization now and in the future.

## Abbreviations

CHASR: Canadian Hub for Applied and Social Research
*r_pb_*: point-biserial correlation coefficient
*r_s_*: Spearman rank correlation coefficient
